# Supplementary material for: Ab initio predictions for polarized deuterium-tritium thermonuclear fusion
Source: Nat Commun. 2019 Jan 21;10:351. doi: 10.1038/s41467-018-08052-6 (PMC6341121; doi:10.1038/s41467-018-08052-6)
Supplement: Supplementary file 1 — Supplementary Information [file 41467_2018_8052_MOESM1_ESM.pdf]

Supplementary Information

Ab initio predictions for polarized deuterium-tritium thermonuclear fusion

Hupin et al.

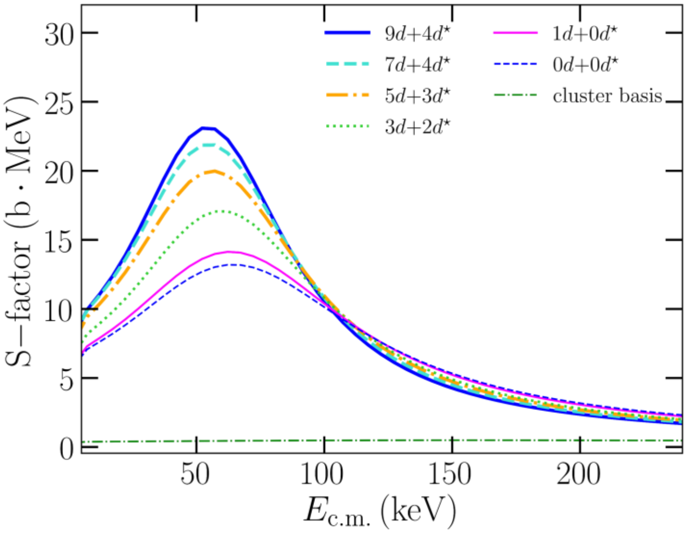

Supplementary Figure 1 Convergence of the DT S-factor with increasing number of positive-energy eigenstates of the deuterium. Results obtained within the NCSMC approach at the harmonic oscillator model-space size $N_{\max} = 11$ plotted as a function of the energy in the center-of-mass frame, $E_{\mathrm{c.m.}}$. The '*nd*+*md**' labels stand for the S-factors obtained by including the first *n* positive-energy eigenstates in the ${}^3S_1$-${}^3D_1$ channel plus the first *m* eigenstates in the ${}^3D_2$ channel of ^{2}H. Results obtained within the cluster basis alone (with the deuterium ground state only) are shown as reference and are labeled as 'cluster basis'.

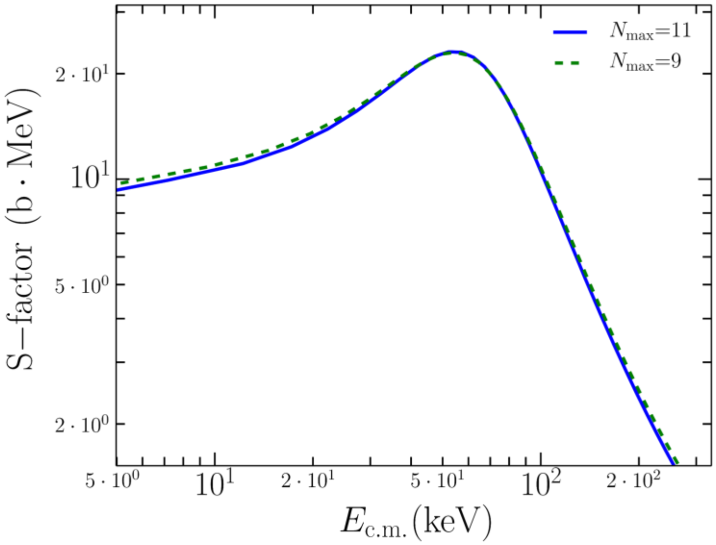

Supplementary Figure 2 Convergence of the DT S-factor with increasing harmonic-oscillator model-space size. The labels '$N_{\max} = 9$' and '$N_{\max} = 11$' stand for the DT S-factors obtained within the NCSMC approach at $N_{\max} = 9$, and 11 (currently the largest achievable). In the figure, $E_{\mathrm{c.m.}}$ is the energy in the center-of-mass frame.

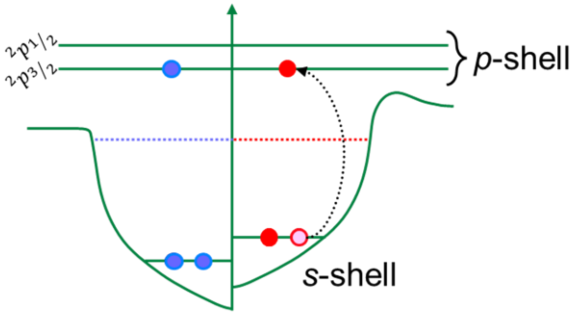

Supplementary Figure 3 Simple sketch of the structure nature of the DT $3/2^+$ resonance. Neutrons are in blue and protons in red.

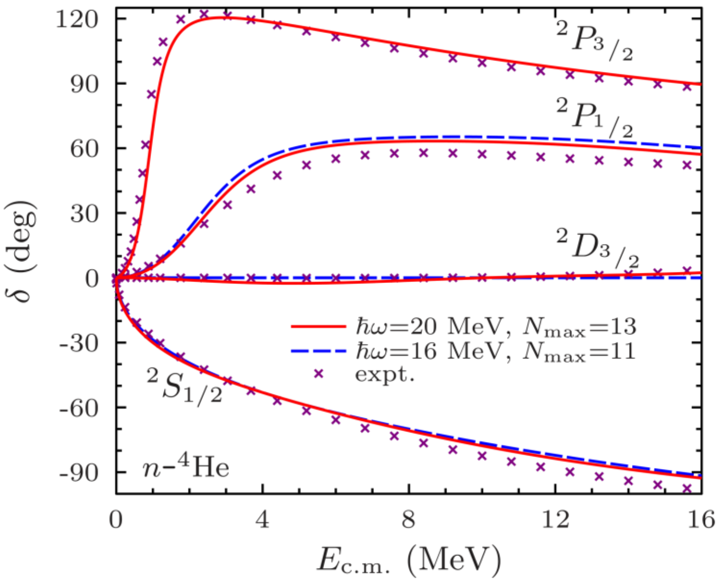

Supplementary Figure 4 Neutron-^{4}He phase shifts below the fusion reaction threshold. Results in the $^2S_{1/2}$, $^2P_{1/2}$, $^2P_{3/2}$, and $^2S_{3/2}$ partial waves obtained in the NCSMC model space without D+T cluster states. The labels '$\hbar\omega = 20$ MeV, $N_{\rm max} = 13$' and '$\hbar\omega = 16$ MeV, $N_{\rm max} = 11$' stand, respectively, for the phase shifts obtained using the $\hbar\omega = 20$ MeV, $\Lambda_{\rm SRG} = 2.0$ fm^{-1}, $N_{\rm max} = 13$ and $\hbar\omega = 16$ MeV, $\Lambda_{\rm SRG} = 1.7$ fm^{-1}, $N_{\rm max} = 11$ sets of NCSMC parameters. An accurate R-matrix parametrization of experimental data (G. M. Hale, personal communication[1]) is shown as a reference, labelled as 'expt.'.

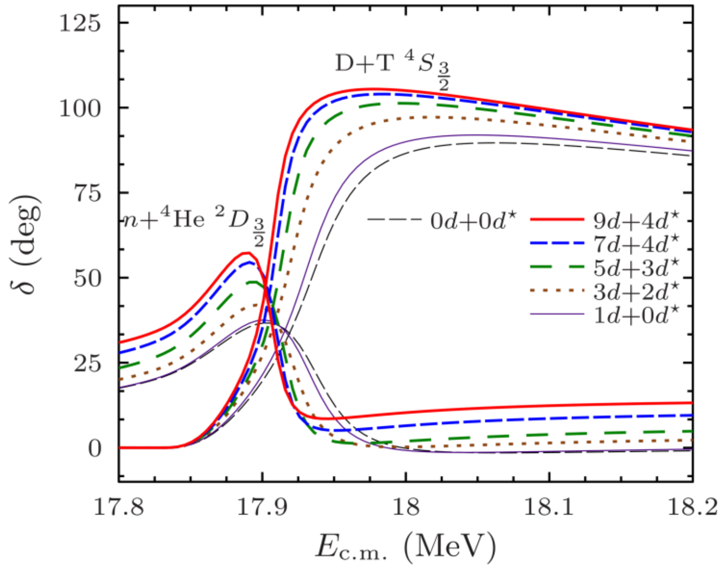

Supplementary Figure 5 Diagonal phase shifts in the entrance and exit channels of the DT fusion. Convergence of the (real part of the) D+T $^4S_{3/2}$ and n+^{4}He $^2D_{3/2}$ diagonal phase shifts (characterizing, respectively, the entrance and exit scattering states of the DT fusion reaction) in the $J^\pi = 3/2^+$ channel with increasing number of positive-energy eigenstates of the deuterium, as obtained within the NCSMC approach. The '*nd*+*md**' labels stand for the phase shifts obtained by including the first *n* positive-energy eigenstates in the $^3S_1$-$^3D_1$ channel plus the first *m* eigenstates in the $^3D_2$ channel of ^{2}H.

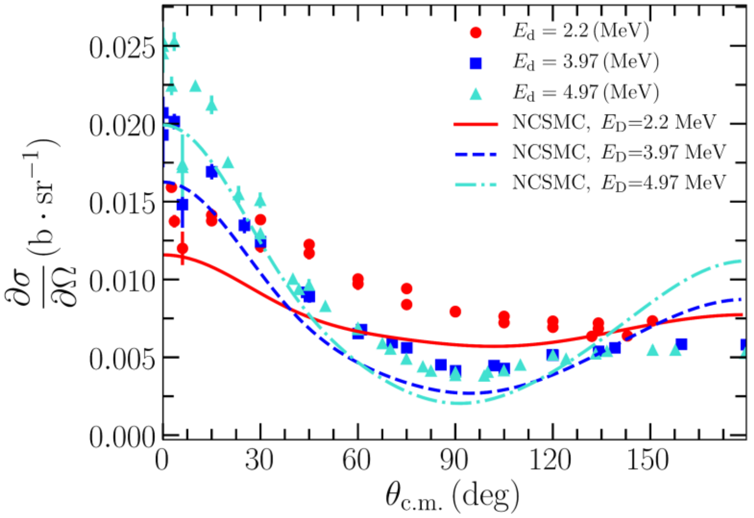

Supplementary Figure 6 Unpolarized differential cross section. Comparison of computed and measured[2-6] differential cross sections $\left(\frac{\partial\sigma}{\partial\Omega}\right)$ in the center-of-mass (c.m.) frame at the deuterium incident energies of $E_{\rm D} = 2.2, 3.97$ and $E_{\rm D} = 4.97$ MeV, as a function of the scattering angle in the c.m. frame, $\theta_{\rm c.m.}$. The labels 'NCSMC' and 'Expt.' stand, respectively for the present results and the experimental data.

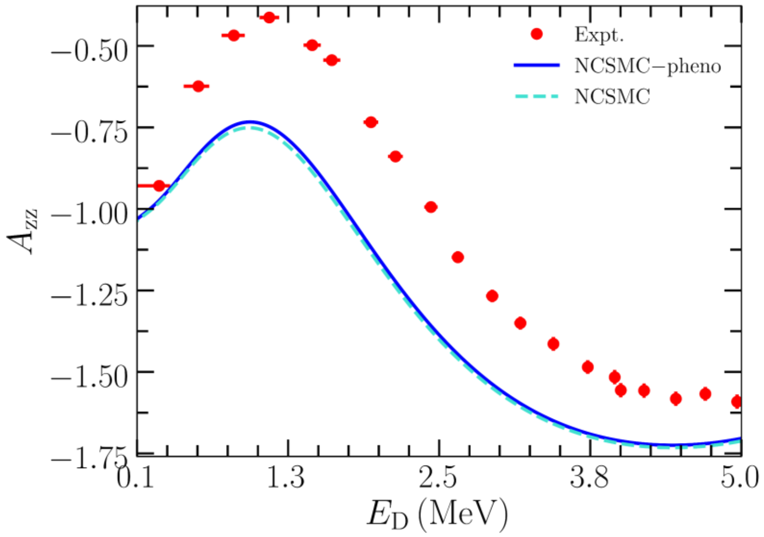

Supplementary Figure 7 Tensor analyzing power for the DT reaction. Comparison between present results and measurements[7] for the tensor analyzing power ($A_{zz}^{(b)}$) at energies below the breakup threshold and center-of-mass angle $\theta_{c.m.} = 0°$ as a function of the deuterium incident energies of E_D. The labels 'Expt.', 'NCSMC', and 'NCSMC-pheno' stand, respectively, for the experimental data, the present calculation, and the results of the present calculation after a phenomenological correction of -5 keV to the position of the $3/2^+$ resonance.

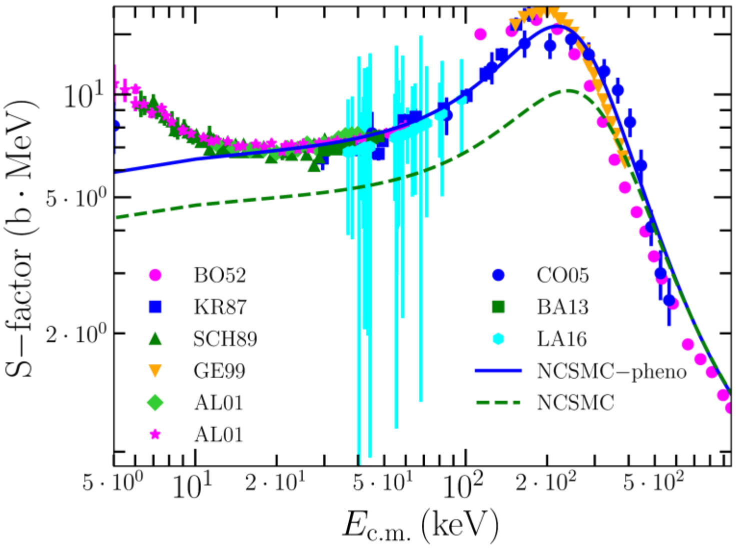

Supplementary Figure 8 D^3He astrophysical S-factor. The results of the present calculation before ('NCSMC') and after ('NCSMC-pheno') phenomenological adjustment of the $3/2^+$ resonance are compared with the experimental data of refs. 8-15 (labelled, in order, 'BO52', 'KR87', 'SCH89', 'GE99', 'AL01', 'CO05', 'BA13', and 'LA16'). In the calculations, the harmonic oscillator model space size is limited to $N_{\max} = 9$ for computational reasons. $E_{c.m.}$ denotes the energy in the center-of-mass frame.

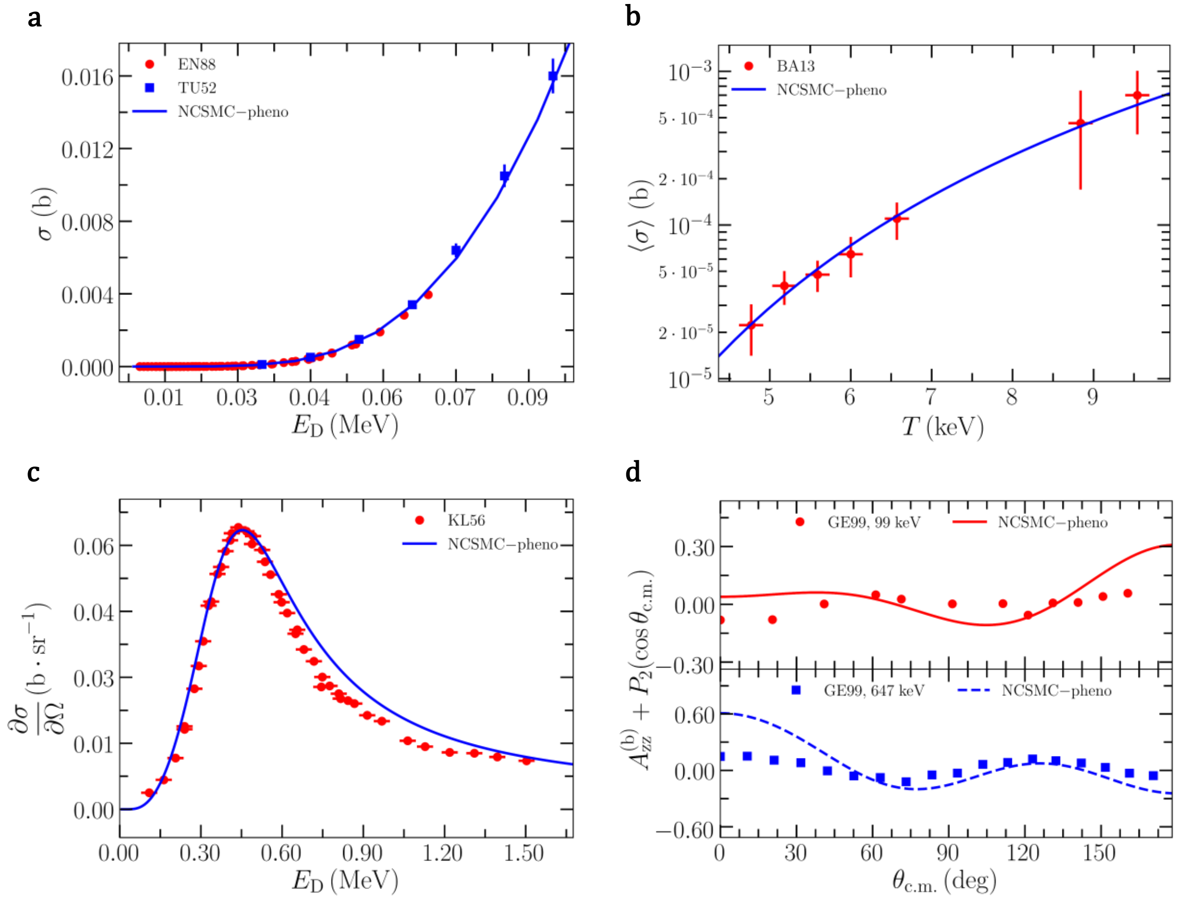

Supplementary Figure 9 D^3He reaction observables. **a** Fusion cross section (σ_{tot}) in the energy range below the resonant peak compared to the measurements of refs. 16 ('TU52') and 17 ('EN88'). **b** Computed temperature averaged cross section ($\langle\sigma\rangle$) compared to the data of ref. 14 ('BA13'). **c** Computed differential cross section $\left(\frac{\partial\sigma}{\partial\Omega}\right)$ at the center-of-mass (c.m.) scattering angle of $\theta_{\text{c.m.}} = 90°$ as a function of the impinging deuterium energy (E_D) compared to the experimental data of ref. 18 ('KL56'). **d** Computed $A_{zz}^{(b)}$ tensor analyzing power after subtraction of the $J^\pi = 3/2^+, \ell = 0$ contribution [given by the Legendre polynomial $-P_2(\cos\theta_{\text{c.m.}})$] compared to the data from ref. 11 ('GE99') at the D incident energies of E_D = 99 keV (top) and 641 keV (bottom). In the figures 'NCSMC-pheno' stands for the results of the present calculations after phenomenological adjustment of the $3/2^+$ resonance.

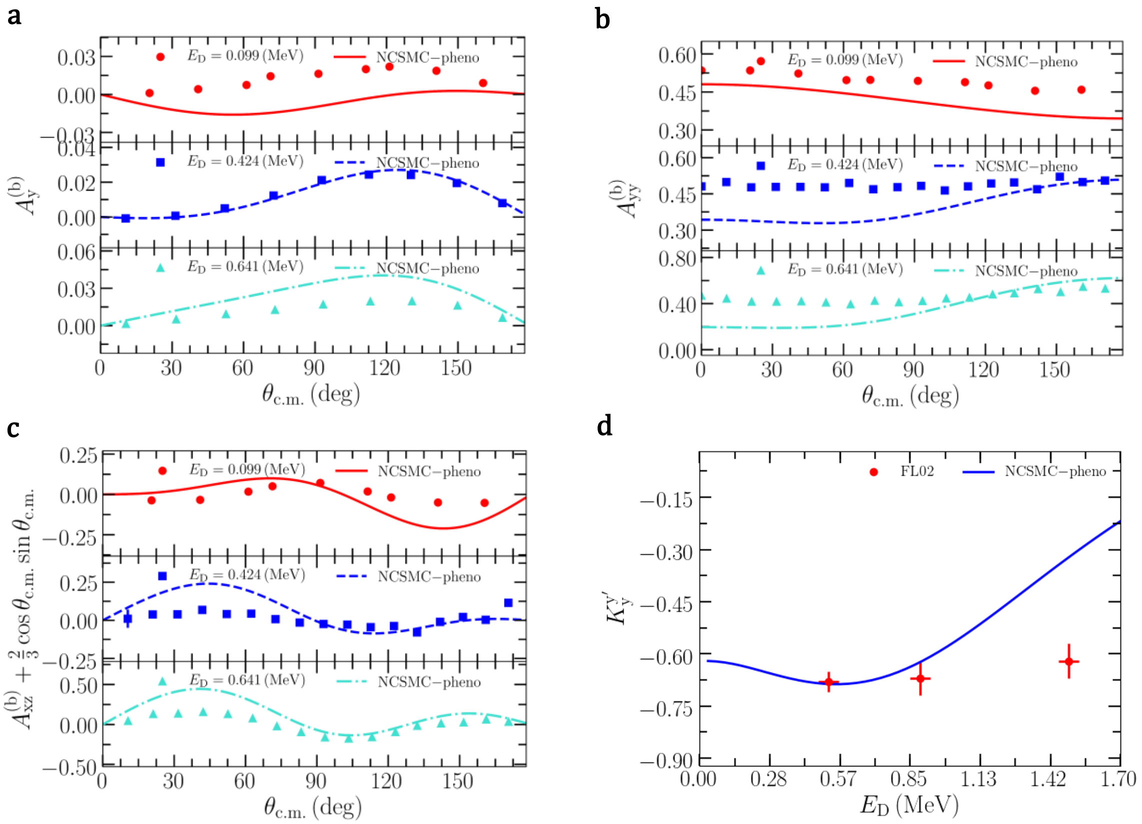

Supplementary Figure 10 D^3He polarization observables. **a-c** Computed $A_y^{(b)}$ vector and $A_{yy}^{(b)}$ and $A_{xz}^{(b)}$ tensor analyzing powers compared to the data from ref. 11 (circles, squares, and triangles) at the D incident energies of E_D = 99 keV (top), E_D = 424 keV (middle), and 641 keV (bottom). The $J^\pi = 3/2^+, \ell = 0$ contribution (given by the Legendre polynomial $-\frac{2}{3}\cos\theta_{c.m.}\sin\theta_{c.m.}$) is subtracted in panel **c**. **d** Computed polarization transfer coefficient ($K_y^{y'}$) around the reaction threshold at the center-of mass angle of $\theta_{c.m.} = 0°$ compared to the experimental data of ref. 19 (circles). In the figures 'NCSMC-pheno' stands for the results of the present calculations after phenomenological adjustment of the $3/2^+$ resonance.

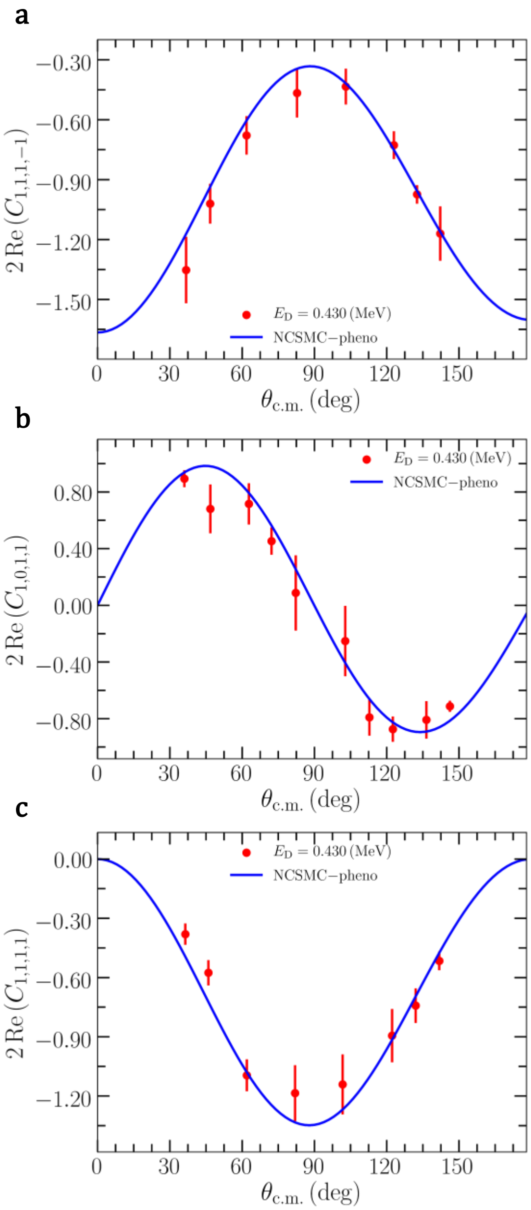

Supplementary Figure 11 D^3He spin correlation coefficients. Computed $C_{1,1,1,-1}$ (**a**), $C_{1,0,1,1}$ (**b**) and $C_{1,1,1,1}$ (**c**) spherical spin correlation coefficients as a function of the center-of mass angle, $\theta_{\mathrm{c.m.}}$, compared to the experimental data from ref. 20 (circles) at the D incident energies of $E_{\mathrm{D}} = 430$ keV. In the figures 'NCSMC-pheno' stands for the results of the present calculations after phenomenological adjustment of the $3/2^+$ resonance.

Supplementary Table 1 ^{5}He $3/2^+$ resonance. Centroid and width of the $3/2^+$ resonance derived from the DT eigenphase shifts computed within the cluster basis alone ('Cluster basis'), and the full NCSMC basis before ('NCSMC)' and after ('NCSMC-pheno') a phenomenological correction of -5 keV to the position of the $3/2^+$ resonance. The centroid is defined as the energy for which the first derivative of the eigenphase shifts is maximal, while the width is computed as twice the inverse of the derivative of the eigenphase shift at the resonance energy. Values derived from an *R*-matrix analysis of experimental data are shown as reference[21].

^{5}He$(3/2^+)$	Cluster basis (D g.s. only)	Cluster basis	NCSMC (D g.s. only)	NCSMC	NCSMC-pheno	*R*-matrix
E_r (keV)	105	120	65	55	50	47
Γ_r (keV)	1100	570	160	110	98	74

Supplementary Table 2 Convergence of the ^{5}He $(3/2^+)$ eigenenergy. Relative difference with respect to the extrapolated infinite model space result of the eigenvalue of the ^{5}He $(3/2^+)$ resonance computed within the NCSM approach as function of the harmonic oscillator (HO) basis size $N_{\max}$. The notation '$\hbar\omega$' and '$\Lambda_{\rm SRG}$' denote, respectively, the HO frequency and resolution scale of the similarity renormalization group transformation adopted in the two sets of displayed calculations.

$N_{\max}$	$\hbar\omega$=20 MeV, $\Lambda_{\rm SRG}$=2.0 fm^{-1}	$\hbar\omega$=16 MeV, $\Lambda_{\rm SRG}$=1.7 fm^{-1}
7	78.70%	42.29%
9	45.04%	18.85%
11	25.68%	8.41%
13	13.78%	-

Supplementary Note 1: Convergence of the calculation

Achieving convergence with respect to the number of eigenstates of the aggregate ^{5}He system is straightforward. All eigenstates in a large range of energies around the region of interest for the deuterium-tritium (DT) fusion can be effortlessly included. The second $3/2^+$ eigenstate is exceptionally impactful. This can be clearly seen in Supplementary Figure 1 by comparing the S-factor (dominated by the $3/2^+$ component of the wave function) computed within the full no-core shell model with continuum (NCSMC) model space with the results obtained within the cluster basis alone. Evidently, the configuration where all five nucleons are in close contact plays an essential role.

Supplementary Figure 1 also shows the somewhat slow but steady convergence pattern of the S-factor with respect to the number (in order of increasing energy) of positive-energy eigenstates of the D projectile included in addition to the (negative-energy) ground state. We used the notation *d*

and d^* for the eigenstates in the ${}^3S_1$-${}^3D_1$ and ${}^3D_2$ channels of ^{2}H, respectively. Since the number of available positive-energy eigenstates of the D projectile depends on the harmonic oscillator (HO) basis size N_{max} (higher N_{max} means more states to discretize the ^{2}H continuum), it is instructive to compare this figure with the convergence in N_{max} (Supplementary Figure 2). The agreement between the $N_{\text{max}} = 9$ and 11 calculations (both including the maximum number of available deuteron states) is quite good.

It should be noted that the S-factor is extremely sensitive to changes in the position of the $3/2^+$ resonance, acting as a magnifying glass. For example, the 35 keV shift to lower energies in the resonance position between the results obtained within the cluster basis alone and the full NCMC model space (see Supplementary Table 1) results in about one order of magnitude increase of the S-factor amplitude. In comparison, the positive-energy eigenstates of the D projectile contribute a shift of 10 keV (15 keV) in the $3/2^+$ resonance centroid when the eigenstates of the aggregate ^{5}He system are (are not) included in the model space. This suggests that there is a strong similarity between some cluster basis states built from the discretization of the D energy continuum and some static solutions of the ^{5}He aggregate. In this contest, it becomes also clear that the fine-tuning of the $3/2^+$resonance centroid required to accurately reproduce the experimental S-factor is extremely challenging, given remaining inaccuracies of the adopted chiral interactions for *p*-shell nuclei. For this reason, we opted for a phenomenological fine tuning as explained in the Methods section.

The $3/2^+$ resonance, which drives the massive enhancement of the S-factor, schematically consists in a proton promoted from the *s*-shell onto the ${}^2p_{3/2}$ sub-shell, as shown in the sketch of Supplementary Figure 3. The energy required is of the order of the splitting between major HO *s*- and *p*-shells and can be inferred from the experimental spectra as approximatively the excitation energy of the $3/2^+$ resonance, that is 16.84 MeV. This explains why the HO frequency of $\hbar\omega =$ 16 MeV chosen in this work contributes to speeding up the convergence of our calculations. Additionally, to ensure the convergence of our calculation within the computationally achievable largest model space ($N_{\text{max}} = 11$), we used a similarity renormalization group (SRG) resolution scale of $\Lambda_{\text{SRG}} = 1.7\ \text{fm}^{-1}$. To further analyze our parameters' choice, in Supplementary Figure 4 we compare the present n + ^{4}He elastic scattering phase shifts (obtained in the NCSMC model space without D+T cluster states) with those previously obtained for $\hbar\omega = 20$ MeV, $\Lambda_{\text{SRG}} =$ $2.0\ \text{fm}^{-1}$ and $N_{\text{max}} = 13$[22,23]. We can see that the agreement is excellent. The fast convergence rate achieved within the present choice of parameters is even more manifest when analyzing the dependence on N_{max} of the ^{5}He $3/2^+$ eigenenergy computed within the NCSM. This is illustrated in Supplementary Table 2, where we show the difference between the computed eigenenergy at a given N_{max} relative to the extrapolated energy at $N_{\text{max}} \longrightarrow \infty$. The frequency closer to the major HO shell splitting performs much better. At $N_{\text{max}} = 11$ the relative difference goes down from 25.68% at $\hbar\omega = 20$ MeV, $\Lambda_{\text{SRG}} = 2.0\ \text{fm}^{-1}$ to 8.41% at $\hbar\omega = 16$ MeV, $\Lambda_{\text{SRG}} = 1.7\ \text{fm}^{-1}$, more than a factor of two.

Supplementary Note 2: Reaction Mechanism

The centroid and width of the $3/2^+$resonance computed within the NCSMC-pheno (i.e., by applying the phenomenological correction of -5 keV to the position of the $3/2^+$ resonance discussed in the Methods) are in good agreement with those extracted from the R-matrix analysis of data of ref. 21, particularly considering that the latter values were obtained from the S-matrix pole rather than from the eigenphase shift, as done here. In addition, the magnitude and width of the computed total n-^{4}He cross section in the energy region of the $3/2^+$resonance are in a good agreement with the measurements of Haesner et al.[24], though the position of its peak is overestimated by about 1%. This is due in part to a slight difference between computed (17.8 MeV) and experimental (17.6 MeV) Q-value, and more in general to remaining inaccuracies of the adopted chiral interactions for p-shell nuclei which cannot be entirely corrected using the minimal (single-parameter) phenomenological adjustment adopted in this work. In Supplementary Figure 5, we show the (real part of the) phase shifts extracted from the diagonal elements of the S-matrix in the $J^\pi = 3/2^+$channel, namely the d-^{3}H $^4S_{3/2}$ and n-^{4}He $^2D_{3/2}$ partial waves belonging, respectively, to the entrance and exit channels of the reaction. Similar to our more limited work of ref. 25, we find a sharp resonant behavior in the d-^{3}H $^4S_{3/2}$ partial wave, but the n-^{4}He $^2D_{3/2}$ phase shift is broader and does not cross 90°. Supplementary Figure 5 also highlights the influence of the deuterium continuum. We would like to stress that this reaction mechanism highlights the fundamental role played by the tensor force, present in both nucleon-nucleon (NN) and three-nucleon (3N) components of the nuclear interactions. In addition, the important role of the 3N force in reproducing the position of the resonance centroids and the splitting of the $^2p_{3/2}$ and $^2p_{1/2}$ sub p-shell levels has also become evident in the last decade[23,26]. Because of this (and the fact that the SRG procedure we use to accelerate the convergence generates induced 3N forces) the inclusion of 3N forces was essential to achieving the present accurate results for the DT fusion.

Supplementary Note 3: Comparison to higher energy data

Our approach is presently valid up to the threshold of the dissociation of the deuterium projectile or 2.224 MeV. Above such energy, it represents an approximation. Nevertheless, in Supplementary Figures 6 and 7 we present a comparison with higher-energy data for the angular differential cross section and tensor analyzing power, respectively. At the deuteron energy of 2.2 MeV and above, the *ab initio* angular differential cross section systematically underestimates the data[2-6], though the shape of the angular distribution is qualitatively reproduced. The tensor analyzing power (presented in Supplementary Figure 7), which is inversely proportional to the differential cross section, further magnifies the difference between theory and experiment. At the origin of this discrepancy are higher-energy ^{5}He resonances (known experimentally and in evaluations[27]) that come into play a few MeV above the peak-energy of the DT fusion, due to the population of the nearby $^2p_{1/2}$ subshell (see the sketch of Supplementary Figure 3). Lacking an exact treatment of three-cluster dynamics and given remaining inaccuracies of the adopted chiral Hamiltonian in reproducing the p-subshell ordering that affects the underlying phase shifts, these resonances are not reproduced with the required level of accuracy (within ~10 keV). We note that this only affects the cross section

at higher energies. In particular, around the energy of interest for fusion applications (~100 keV), the tensor analyzing power is in good agreement with experiment.

Supplementary Note 4: Calculation for the mirror D^3He reaction

In Supplementary Figure 8, we compare our computed D^3He S-factor and its phenomenological correction to available data[8-15]. All parameters of the NCSMC calculation match those of the used for the DT reaction (see Supplementary Note 1) but the N_{max} value, which in this case is limited to nine major shells for computational reasons. As in the DT case, the centroid position of the $3/2^+$ resonance of ^5Li is overestimated and needs to be corrected phenomenologically. Once again, the adjustment of the $3/2^+$ resonance is strongly constrained by reproducing the S-factor from ~20 keV to energies below the resonance. At lower energies (below ~20 keV), the prediction is expected to disagree with data due to laboratory electron screening effects, which enhance the cross section masking the ("bare") nuclear S-factor. At the peak of the S-factor, the experimental picture is somewhat uncertain. Our results are in good agreement with the data of ref. 13 ('CO05'). The computed peak value of the reaction cross section (798 mb) is in good agreement with the experimental value of 777 ± 33 mb reported by Geist et al.[11] ('GE99'). However, the position of the peak is found at 450 keV, 24 keV above the energy reported in ref. 11. This slight energy shift is at the origin of the discrepancy between our calculation and the S-factor of Geist et al. In the present (restricted) $N_{\text{max}} = 9$ model space, a 426 keV peak energy is inconsistent with the behavior of the S-factor at lower energy. There, we find good agreement with the total reaction cross section and thermalized cross section data of refs. 16, 17 and 14, respectively, owing to the tight constraint imposed by this energy regime on our phenomenological adjustment (see Supplementary Figures 9a and 9b). In Supplementary Figure 9c we compare the computed differential cross section at $\theta_{\text{c.m.}} = 90°$ with the experimental data up to $E_D = 1.6$ MeV of Klucharev et al.[18]. Overall, a small overestimation of data is noticeable above the fusion peak suggesting once again that the width of the $3/2^+$ resonance may be slightly overestimated. Based on the trend shown by the DT results of Supplementary Figure 2, we expect that an $N_{\text{max}} = 11$ calculation would yield a narrower cross section peak in closer agreement with the experimental data of both Geist et al and Klucharev et al. We also computed an array of polarization observables, namely the vector $A_{\text{y}}^{(\text{b})}$ and tensor $A_{\text{zz}}^{(\text{b})}$, $A_{\text{yy}}^{(\text{b})}$, and $A_{\text{xz}}^{(\text{b})}$ analyzing powers at the deuteron incident energies of 99, 424 and 641 keV, and the polarization transfer coefficient $K_{\text{y}}^{\text{y}'}$ at $\theta_{\text{c.m.}} = 0°$, to compare to the measurements reported in refs. 11 and 19, respectively. The comparisons for the analyzing powers are shown in Fig. 3a of the Results section, Supplementary Figures 9d, and 10a-c, while that for the polarization transfer coefficient is presented in Supplementary Figure 10d. In general, we find fairly good agreement with the experimental data at 424 keV, while at the left and right of the peak we tend to obtain a good description of the overall angular and energy dependence but somewhat overestimate the amplitude. This can be traced back to the modest overestimation of the reaction cross section. Finally, in Supplementary Figure 11 we compare our computed spin correlation coefficients $C_{1,1,1,-1}$, $C_{1,0,1,1}$ and $C_{1,1,1,1}$ to the experimental data of ref. 20, at the incident deuteron energy of 430 keV. This is the only existing spin-correlation experiment in the resonance region for this reaction, and the most significant and direct test of our calculations for the polarized fusion. In our

notation, the coefficients are written as spherical tensors with the initial (final) two indices corresponding to the rank and projection of the tensor moments of the beam (target). Our calculation agrees well with the experimental data. This stands as a chief validation of our predictions for the polarized DT fusion. Overall, our *ab initio* method together with modern chiral NN+3N interactions are able to reproduce both the DT fusion and its mirror D^3He reaction. This is a major step forward compared to the results obtained in our earlier work[25].

Supplementary Discussion: Uncertainties of the calculation

In the present work, uncertainties derive either from the many-body model used to solve the five-body Schrödinger equation or from the employed nuclear Hamiltonian. The formers are addressed in Supplementary Note 1. There, we show that our calculation is converged with respect to the three parameters $\hbar\omega, N_{\max}$ and Λ_{SRG}. Thus, uncertainties from the many-body technique are particularly small, typically close to the size of the line width as exemplified in Fig. 6 of the Results section. To demonstrate that the accuracy of the present application is not accidental, we computed the D^3He mirror reaction and compared both unpolarized and polarized reaction observables to data. We obtained satisfactory agreement with data that further validates our predictions for the DT polarized observables. On the other hand, it is computationally extremely challenging for the time being to give an estimate of the uncertainties pertaining the nuclear Hamiltonian. We use a chiral EFT Hamiltonian that has been proven to reproduce properties of the $A = 3, 4, 5, 6$ nuclei, including *p*-shell physics. Based on the fact that other chiral EFT Hamiltonians have emerged that fail to reproduce the low-lying *p*-waves of the ^5He system, it is expected that uncertainties from the nuclear interaction model may be significant, but this remains to be investigated.

Supplementary References

1. Hale, G.M., ENDF/B-VII.1 MAT 228, September 2010.
2. Otuka, N., et al. Nuclear Data Sheets **120**, 272-276 (2014).
3. Galonsky, A., Johnson, C.H., Cross Sections for the $T(d,n)^4He$ Reaction. *Phys. Rev.* **104**, 421-425 (1956).
4. Bame, S.J. Jr., Perry, J.E. Jr., $T(d,n)^4He$ Reaction. *Phys. Rev.* **107**, 1616-1620 (1957).
5. McDaniels, D.K., Drosg, M., Hopkins, J.C., Seagrave, J.D., Angular Distributions and Absolute Cross Sections for the $T(d,n)^4He$ Neutron-Source Reaction. *Phys. Rev. C* **7**, 882-888 (1973).
6. Liskien, H., Paulsen, A., Neutron production cross sections and energies for the reactions $T(p,n)^3He$, $D(d,n)^3He$, and $T(d,n)^4He$. *Nucl. Data Tables* **11**, 569-619 (1973).
7. Dries, L.J., Clark, H.W., Detomo R. Jr., Regner, J.L., Donoghue, T.R., $A_{zz}(0°)$ for the charge-symmetric $^3He(\vec{d},p)^4He$ and $^3H(\vec{d},n)^4He$ reactions below 6.75 MeV. *Phys. Rev. C* **21**, 475-482 (1980).
8. Bonner, T.W., Conner, J.P., Lillie, A.B., Cross Section and Angular Distribution of the $^3He(d,p)^4He$. *Phys. Rev.* **88**, 473-476 (1952).
9. Krauss, A., Becker, H.W., Trautvetter, H.P., Rolfs, C., Brand, K., Low-energy fusion cross sections of D+D and $D+^3He$ reactions. *Nucl. Phys. A* **465**, 150-172 (1987).

10. Schröder, U., Engstler, S., Krauss, A., Neldner, K., Rolfs, C., Somorjai, E., Langanke, K., Search for electron screening of nuclear reactions at sub-coulomb energies. *Nucl. Instrum. Methods Phys. Res. B* **40–41**, 466-469 (1989).
11. Geist, W.H., Brune, C.R., Karwowski, H.J., Ludwig, E.J., Veal, K.D., Hale, G.M., The ^{3}He($\vec{d}$,p)^{4}He reaction at low energies. *Phys. Rev. C* **60**, 054003 (1999).
12. Aliotta, M., et al., Electron screening effect in the reactions ^{3}He(d,p)^{4}He and d(^{3}He,p)^{4}He. *Nucl. Phys. A* **690**, 790-800 (2001).
13. La Cognata, M., et al., Bare-nucleus astrophysical factor of the ^{3}He(d,p)^{4}He reaction via the "Trojan horse" method. *Phys. Rev. C* **72**, 065802 (2005).
14. Barbui, M., et al., Measurement of the Plasma Astrophysical *S* Factor for the ^{3}He(d,p)^{4}He Reaction in Exploding Molecular Clusters. *Phys. Rev. Lett.* **111**, 082502 (2013).
15. Lattuada, D., et al., Model-independent determination of the astrophysical S factor in laser-induced fusion plasmas. *Phys. Rev. C* **83**, 045808 (2016).
16. Arnold, W.R., Phillips, J.A., Sawyer, G.A., Stovall, E.J., Tuck, J.L., Cross Sections for the Reactions D(d,p)T, D(d,n)^{3}He, T(d,n)^{4}He, and ^{3}He(d,p)^{4}He below 120 keV. *Phys. Rev.* **93**, 483-497 (1954).
17. Engstler, S., Krauss, A., Neldner, K., Rolfs, C., Schröder, U., Langanke, K., Effects of electron screening on the ^{3}He(d,p)^{4}He low-energy cross sections. *Phys. Lett. B* **202**, 179-184 (1988).
18. Klucharev, A.P., Eselśon, B.N., Valťer, A.K., Study of ^{3}He reaction with deuterons. *Sov. Phys.-Doklady* **1**, 475 (1956).
19. Fletcher, K.A., et al., $K_y^{y'}(0°)$ for ^{3}He(d,p)^{4}He near the $J^\pi = \frac{3}{2}^+$ resonance. *Phys. Rev. C* **66**, 057601 (2002).
20. Leemann, Ch., Bürgisser, H., Huber, P., Rohrer, U., Paetz gen. Schieck, H., Seiler, F., Die ^{3}He(d,p)^{4}He Reaktion mit polarisiertem und unpolarisiertem Target und polarisiertem Deuteronenstrahl bei E_d = 430 keV. *Helv. Phys. Acta* **44**, 141-159 (1971); *Ann. Phys.* **66**, 810-815 (1971).
21. Hale, G.M., Brown, R.E, Jarmie, N., Pole structure of the $J^\pi = 3/2^+$ resonance in ^{5}He. *Phys. Rev. Lett.* **59**, 763-766 (1987).
22. Navrátil, P., Quaglioni, S., Hupin, G., Romero-Redondo, S., Calci, A., Unified *ab initio* approaches to nuclear structure and reactions. *Phys. Scripta* **91**, 053002 (2016).
23. Hupin, G., Langhammer, J., Navrátil, P., Quaglioni, S., Calci, A., Roth, R., *Ab initio* many-body calculations of nucleon-^{4}He scattering with three-nucleon forces. *Phys. Rev. C* **88**, 054622 (2013).
24. Haesner, B., Heeringa, W., Klages, H.O., Dobiasch, H., Schmalz, G., Schwarz, P., Wilczynski, J., Zeitnitz, B., Measurement of the ^{3}He and ^{4}He total neutron cross sections up to 40 MeV. *Phys. Rev. C* **28**, 995-999 (1983).
25. Navrátil, P., Quaglioni, S., *Ab initio* Many-Body Calculations of the ^{3}H(d,n)^{4}He and ^{3}He(d,p)^{4}He Fusion Reactions. *Phys. Rev. Lett.* **108**, 042503 (2012).
26. Nollett, K.M., Pieper, S.C., Wiringa, R.B., Carlson, J., Hale, G.M., Quantum Monte Carlo Calculations of Neutron-α Scattering. *Phys. Rev. Lett.* **99**, 022502 (2007).
27. Tilley, T.R., Cheves, C.M., Godwin, J.L., Hale, G.M., Hofmann, H.M., Kelley, J.H., Sheu C.G., Weller, H.R., Energy levels of light nuclei A=5, 6, 7. *Nucl. Phys. A* **708**, 3-163 (2002).
